# Phylogeny, Taxonomy, and Biogeography of *Pterocarya* (Juglandaceae)

**DOI:** 10.3390/plants9111524

**Published:** 2020-11-09

**Authors:** Yi-Gang Song, Ying Li, Hong-Hu Meng, Yann Fragnière, Bin-Jie Ge, Hitoshi Sakio, Hamed Yousefzadeh, Sébastien Bétrisey, Gregor Kozlowski

**Affiliations:** 1 Eastern China Conservation Center for Wild Endangered Plant Resources, Shanghai Chenshan Botanical Garden, Chenhua Road No.3888, Songjiang, Shanghai 201602, China; ying909726271@sina.com (Y.L.); gebinjie123@163.com (B.-J.G.); 2 Department of Biology and Botanic Garden, University of Fribourg, Chemin du Musée 10, CH-1700 Fribourg, Switzerland; yann.fragniere@unifr.ch (Y.F.); sebastien.betrisey@unifr.ch (S.B.); 3 Shanghai Chenshan Plant Science Research Center, Chinese Academy of Sciences, Chenhua Road No.3888, Songjiang, Shanghai 201602, China; 4 Centre for Integrative Conservation, Xishuangbanna Tropical Botanical Garden, Chinese Academy of Sciences, Xuefu Rd. 88, Wuhua, Kunming 650223, China; menghonghu@xtbg.ac.cn; 5 Field Center for Sustainable Agriculture and Forestry, Faculty of Agriculture, Niigata University, Sado-city, Niigata 950-2181, Japan; sakiohit@gmail.com; 6 Faculty of Natural Resources, Department of Forestry, Tarbiat Modares University (TMU), Mazandaran 14115-111, Iran; h.yousefzadeh@modares.ac.ir; 7 Natural History Museum Fribourg, Chemin du Musée 6, CH-1700 Fribourg, Switzerland

**Keywords:** divergence time, East Asia-southern Caucasus disjunction, Late Miocene diversification, phylogenomic relationship, refugia, restriction site-associated DNA sequencing (RAD-seq)

## Abstract

Relict species play an important role in understanding the biogeography of intercontinental disjunctions. *Pterocarya* (a relict genus) is the valuable model taxon for studying the biogeography of East Asian versus southern European/West Asian disjunct patterns. This disjunction has not been as well studied as others (e.g., between Eastern Asia and North America). Several phylogenetic studies on *Pterocarya* have been conducted, but none have provided a satisfactory phylogenetic resolution. Here, we report the first well-resolved phylogeny of *Pterocarya* using restriction site-associated DNA sequencing data based on the sampling of all taxa across the entire distribution area of the genus. Taxonomic treatments were also clarified by combining morphological traits. Furthermore, fossil-calibrated phylogeny was used to explore the biogeography of *Pterocarya*. Our results support the existence of two sections in *Pterocarya*, which is in accordance with morphological taxonomy. Section *Platyptera* comprises three species: *P. rhoifolia*, *P. macroptera*, and *P. delavayi*. Section *Pterocarya* also comprises three species: *P. fraxinifolia*, *P. hupehensis*, and *P. stenoptera*. The divergence between the two sections took place during the early Miocene (20.5 Ma). The formation of the Gobi Desert and climate cooling of northern Siberia in the Middle Miocene (15.7 Ma) might have caused the split of the continuous distribution of this genus and the formation of the East Asian versus southern European/West Asian disjunct pattern. Lastly, the divergence between *P. hupehensis* and *P. stenoptera* as well as between *P. rhoifolia* and *P. macroptera*/*P. delavayi* (10.0 Ma) supports the late Miocene diversification hypothesis in East Asia.

## 1. Introduction

Understanding geographic patterns of species diversity is one of the central aims of biogeography [1,2,3]. North temperate disjunctions among East Asia, southern Europe/West Asia, eastern North America, and western North America refugia are certainly the best known and most frequently studied of all the major intercontinental disjunctions [1,2,4,5,6,7]. Among the northern temperate disjunctions, many analyses have been conducted between (1) East Asia and eastern North America [8,9,10,11,12], (2) East Asia and western North America [13,14], (3) eastern North America and western North America [15], (4) southern Europe and western North America [3,16], and (5) of the trans-Atlantic disjunction [17,18]. There is a group of genera (e.g., *Acer*, *Aesculus*, *Forsythia*, *Liquidambar*, *Picea*, *Parrotia*, *Pterocarya*, and *Zelkova*) sharing the East Asian versus southern European/West Asian disjunct pattern, including those in North America [19,20,21,22]. However, all these studies ignore the biogeography of East Asia versus southern European/West Asian disjunct patterns.

Relicts are species that were abundant and occurred in a large area at an earlier geological time, but now only occur in one or a few small areas (so called refugia) [7]. The Sino-Japanese Floristic Region (SJFR) in East Asia harbors the most diverse temperate flora worldwide and is the most important glacial refugium for Cenozoic relict flora [23]. Many of the previous phylogeographic studies in the SJFR focused on individual regions, such as the Sino-Himalayan Forest [24,25,26] and the Sino-Japanese Forest [27,28,29], as well as on a single species. Hence, the expansion of phylogeographic studies to multiple pairs of sister species or groups of closely related taxa has been advocated [23,28,30,31]. More recently, the late Miocene diversification hypothesis was raised, proposing that the Cenozoic relict flora in East Asia split into southern and northern lineages during the late Miocene [1,2,7,30,32]. However, additional relict genera should be studied in detail to test this hypothesis.

*Pterocarya* Kunth (Juglandaceae) is a small Cenozoic relict genus whose species live in riparian areas, with six to eight species [33,34,35]. The members of this genus were widely distributed throughout the Northern Hemisphere during the Miocene, while currently they are limited only to the areas of East Asia and the southern Caucasus (part of West Asia) [36,37,38]. The disjunct distribution between East Asia (five to seven species) and West Asia (one species) makes *Pterocarya* a perfect candidate for the exploration of the evolutionary history of genera with disjunct distribution between East Asia and southwestern Eurasia. Additionally, the main distribution in East Asia provides a chance to test the late Miocene diversification hypothesis.

**Figure 1:**
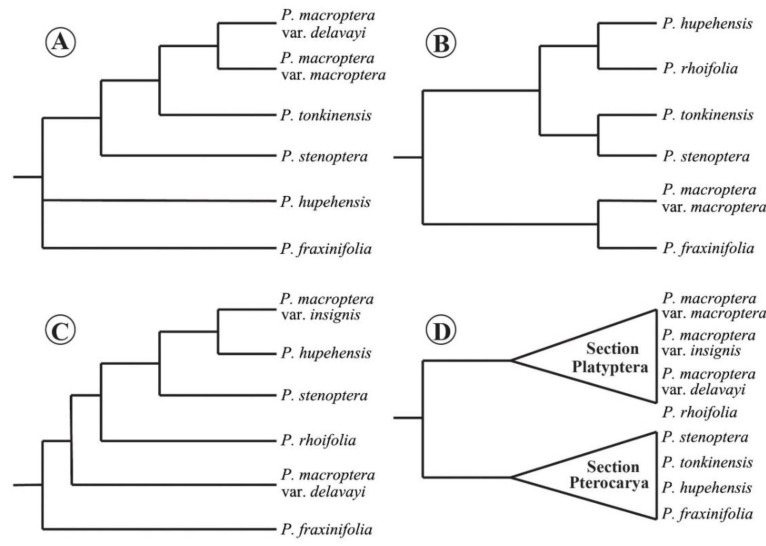
Previous phylogenetic topologies of *Pterocarya* based on different data sets. (**A**) Results based on five chloroplast markers (*rbc*L, *mat*K, *trn*L, *trn*L-F, and *atp*B-*rbc*L) [43]; (**B**) results based on three chloroplast (*rbc*L, *mat*K, and *trn*L-F) and two nuclear loci (ITS and Crabs Claw) [44]; (**C**) results based on nuclear microsatellite and plastid DNA markers [35]; (**D**) two-section classification interpreted as a phylogenetic hypothesis [34].

Molecular phylogeny is an important basic framework for biogeography to study the patterns and processes that shape the distributions of life over a wide range of spatial and temporal scales [12,39,40,41]. Using chloroplast DNA fragments and low-copy nuclear gene data, previous phylogenetic studies on *Pterocarya* have recovered several provisional frameworks within the genus and identified its position within the Juglandaceae [35,42,43,44,45]. The monophyly of *Pterocarya* is strongly supported, whereas phylogenetic relationships among species in the genus are controversial and remain unresolved (Figure 1A–C). In addition, taxon sampling has not adequately addressed issues related to taxonomic treatments for several taxa. Overall, there are three significant conflicts: (1) the traditional division of the genus into two sections (sect. *Platyptera* and sect. *Pterocarya*) is not supported by the current molecular phylogeny; (2) the phylogenetic position of *P. hupehensis* and *P. macroptera* is erratic; and (3) there are still controversies on the taxonomic delimitations among closely related *Pterocarya* species (e.g., three different species mentioned in the Chinese edition of the Flora of China were merged into one taxon (*P. macroptera*), in contrast to the English edition) [33,34] (Figure 1).

In recent years, phylogenomics has provided a more robust phylogenetic framework, and has breathed new life into biogeography [12]. Restriction site-associated DNA sequencing (RAD-seq) produces abundant single-nucleotide polymorphism (SNP) data throughout the genome, which can be used for phylogenetic inference [46,47,48,49]. The RAD-seq approach, in particular, has proven useful in reconstructing fine-scale relationships within closely related species, recently diverged species, and species experiencing interspecific gene flow [41,50,51,52].

The present study aimed to answer the following research questions: (1) What are the stable and well-resolved phylogenetic relationships within the genus *Pterocarya*? (2) What are the taxonomic treatments based on molecular and morphological analyses? (3) Which biogeographic and speciation events could be responsible for the disjunct distribution of the genus between the East Asian and southwestern Eurasian refugia? (4) Did the sections *Platyptera* and *Pterocarya* in the East Asia follow the late Miocene diversification? To address these questions, a comprehensive sample collection strategy was used as well as the following analyses: (1) phylogenetic topology was reconstructed based on RAD-seq data; (2) systematic morphometric analysis was used to clarify taxonomic treatments; and (3) divergence times and biogeographic historical events were estimated based on a fossil-calibrated phylogeny.

## 2. Results

### 2.1. RAD-seq and Data Matrices for Phylogenetic Inference

The Illumina sequencing yielded an average of 11,055,000 reads (raw reads) per sample, ranging from 4,780,000 to 18,580,000. After quality filtering, the average was reduced to 9,947,083 reads (clean reads) per sample, ranging from 3,790,000 to 17,290,000. The sequencing quality was high because the average Q30 was 91.54% per sample, ranging from 88.55% to 92.39%. The mean GC percentage of all the samples was 47.10%, ranging from 43.03% to 58.57% (Table 1). Detailed information concerning the RAD-seq data processing was given in Table S2.

We recovered an average of 5,502,955 reads (RAD tags) after filtering the data de novo via IPYRAD. We obtained 1,728,343 clusters per sample with a mean depth of 15.67. The consensus loci that passed filtering for paralogs ranged from 38,695 to 204,925, and the average was 102,981. The mean sequencing error (E = 0.0103) was lower than the heterozygosity (H = 0.0413). Lastly, the samples had an average of 9287 (ranging from 4222 to 13,650) unlinked SNP sites in the final data sets for phylogenetic inference (Table 2 and Table S2).

### 2.2. RAD-seq Phylogenetic Reconstruction

**Figure 2:**
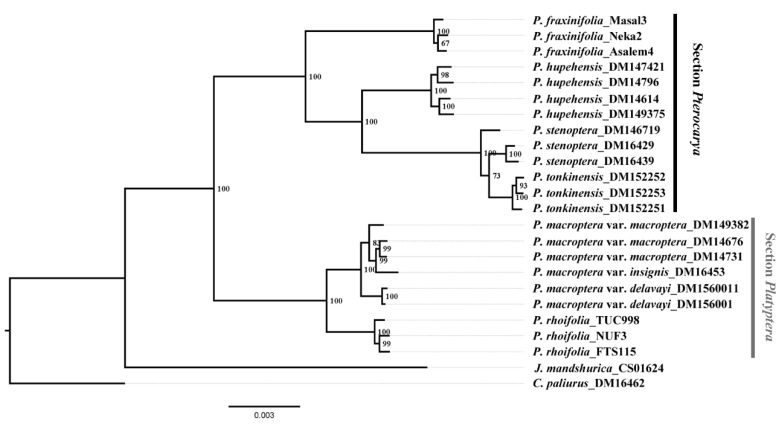
Phylogenetic tree inferred from RAD-seq data for 22 *Pterocarya* individuals and 2 outgroup taxa using RaxML. The numbers next to the nodes of the binary branches are bootstrap values.

Both ML and BI analyses of the final data set showed that the genus *Pterocarya* is monophyletic with two sections: sect. *Pterocarya* (which includes *P. fraxinifolia*, *P. hupehensis*, *P. stenoptera*, and *P. tonkinensis*), and sect. *Platyptera* (which includes *P. rhoifolia* and three varieties of *P. macroptera*) (Figure 2 and Figure S1).

*Pterocarya fraxinifolia* was sister to the other three species in sect. *Pterocarya*. Accessions of *P. stenoptera* were inferred to be paraphyletic, with a population sampled from Zhejiang Province appearing to be more closely related to the accession of *P. tonkinensis* than to the accessions of *P. stenoptera* from Shaanxi Province (Figure 2 and Figure S1). Within sect. *Platyptera*, *P. rhoifolia* was sister to the clades of *P. macroptera*. However, the three varieties of *P. macroptera*, *P. macroptera* var. *delavayi* were inferred to be sister to the other two varieties, with 100% bootstrap (BS) support (Figure 2 and Figure S1).

### 2.3. Morphological Traits and Taxonomic Conclusions

**Figure 3:**
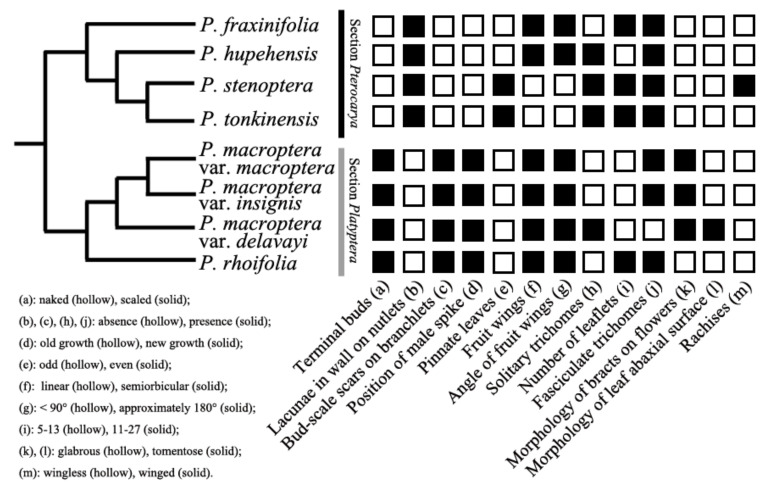
Distribution of taxonomic morphological features in *Pterocarya* based on new phylogenetic tree.

**Figure 4:**
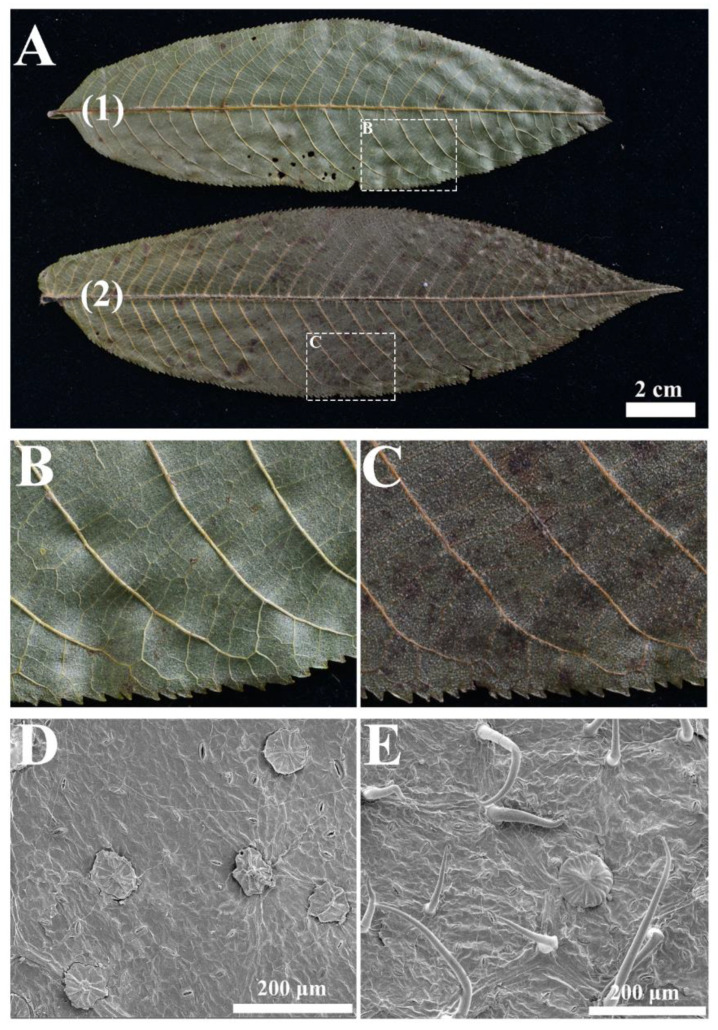
Morphology of leaves of *Pterocarya macroptera* (**A(1)**,**B**,**D**) and *Pterocarya delavayi* (**A(2)**,**C**,**E**).

According to the comparative studies of whole morphologies, together with phylogenetic results of the study, there were four main differences between the two sections: (1) terminal buds, which are either naked (sect. *Pterocarya*) or scaled (sect. *Platyptera*); (2) presence (sect. *Pterocarya*) or absence (sect. *Platyptera*) of lacunae in the walls of the nutlets; (3) presence (sect. *Platyptera*) or absence (sect. *Pterocarya*) of bud-scale scars on branchlets; and (4) the position of male spikes on old growth (sect. *Pterocarya*) or new growth (sect. *Platyptera*). *Pterocarya stenoptera* and *P. tonkinensis* were differentiated only by winged and wingless rachises, respectively (Figure 3). With respect to *P. macroptera*, the mature leaves of the variety *delavayi* substantially differed from the other two varieties (var. *macroptera* and *insignis*), especially regarding the microstructures of the trichomes. The mature leaves of variety *delavayi* exclusively had solitary trichomes, whereas the other two varieties have fasciculate trichomes scattered along the main and secondary veins (Figure 3 and Figure 4).

### 2.4. Estimation of Divergence Times

**Figure 5:**
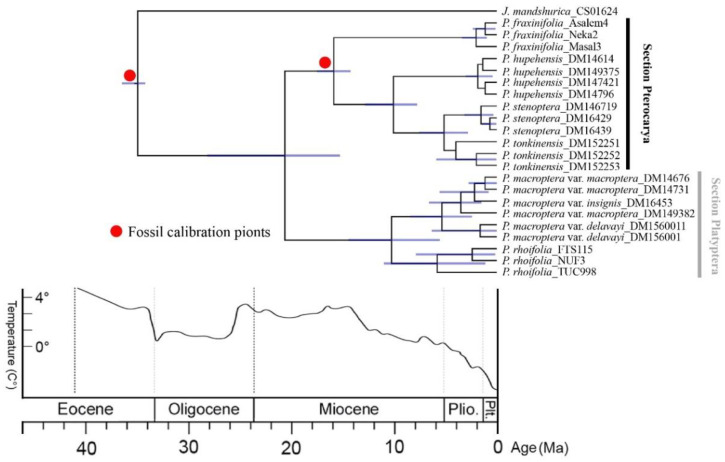
Timing of diversification in *Pterocarya*. Chronogram derived from a MCC tree estimated via the uncorrelated exponential model in BEAST. The blue bars indicate the 95% HPD intervals of the age estimate. Geological time abbreviations: Plio. = Pliocene; Plt. = Pleistocene. The climatic sequence of the major global temperature trends was redrawn from that of [53].

The estimated divergence of *Pterocarya* from *Juglans* was on the order of 34.73 Ma, with 95% highest posterior density (HPD: 34.0–36.2 Ma). The crown age of *Pterocarya* with the divergence of the two sections was 20.48 Ma (early Miocene, 95% HPD: 15.20–27.96 Ma). The estimated crown age of sect. *Pterocarya* with the divergence of *P. fraxinifolia* from the other species of this section was approximately 15.74 Ma (95% HPD: 14.16–17.37 Ma). The split between *P. hupehensis* and the *P. stenoptera*/*P. tonkinensis* clade was estimated to have occurred at 9.98 Ma (95% HPD: 7.73–12.73 Ma). The estimated crown age of sect. *Platyptera* with the divergence of *P. rhoifolia* from *P. macroptera* was approximately 10.17 Ma (95% HPD: 5.51–14.30 Ma), and the split of *P. macroptera* var. *delavayi* from the other two varieties was estimated to have occurred in 5.30 Ma (95% HPD: 2.41–8.37 Ma) (Figure 5).

## 3. Discussion

### 3.1. Phylogenetic Hypothesis for Pterocarya

We presented a robust phylogenetic reconstruction of *Pterocarya* based on RAD-seq data. Although there have been previous efforts to understand the phylogenetic and biogeographical history of *Pterocarya* [35,43,44,45], no study to date has included all six species and all three varieties of *P. macroptera* together in a molecular analysis. According to the molecular phylogeny of Xing et al. (2014) [44] in which three chloroplast loci (*rbc*L, *mat*K, and *trn*L-F) and two nuclear loci (internal transcribed spacer [ITS] and Crabs Claw) were used, *Pterocarya* split into two clades: (1) *P. fraxinifolia* and *P. macroptera* var. *macroptera* clustered into one clade, and (2) the other four species clustered into another clade (Figure 1B). Based on the ITS and *trn*H-*psb*A loci, Mostajeran et al. (2016) [45] proposed three clades within *Pterocarya*: (1) *P. fraxinifolia*, (2) *P. hupehensis* and *P. macroptera*, and (3) *P. stenoptera* and *P. tonkinensis*. Xiang et al. (2014) [43] also proposed three clades based on five chloroplast markers (*rbc*L, *mat*K, *trn*L, *trn*L-F, and *atp*B-*rbc*L): (1) *P. fraxinifolia*; (2) *P. hupehensis*; and (3) *P. stenoptera*, *P. tonkinensis*, *P. macroptera* var. *macroptera*, and *P. macroptera* var. *delavayi* (Figure 1A). Maharramova et al. (2018) [35] suggested that *P. fraxinifolia* is the ancestor of the East Asiatic species (Figure 1C) and that *P. macroptera* var. *insignis* was closely related to *P. hupehensis*, and thus, that it is more distantly related to *P. macroptera* var. *delavayi* (Figure 1C).

Compared with traditional methods, RAD-seq can acquire an abundance of polymorphic markers to solve the problems of few identified gene loci and poor representative genomic information [46,47,48,49,54]. Unlike previous studies, our molecular phylogenetic topology showed 100% support for the separation of the genus *Pterocarya* into two sections (sect. *Pterocarya* and sect. *Platyptera*), which is consistent with the classical taxonomy based on morphological characteristics summarized in Flora of China (FOC) [33,34]. Section *Pterocarya* showed a disjunct distribution between *P. fraxinifolia* in the Caucasus region and the other three species (*P. hupehensis*, *P. stenoptera*, and *P. tonkinensis*) in East Asia, with 100% support. Section *Platyptera*, also with 100% support, comprises two taxa from East Asia: the Japanese endemic *P. rhoifolia* and the Chinese endemic *P. macroptera*. This RAD-seq tree provides a valuable framework for understanding the phylogeny of all species and varieties within the *Pterocarya* genus.

### 3.2. Taxonomic Implications and Evolutionary Importance of Morphological Features

Taxonomy requires an integrative approach to effectively define species boundaries [55]. Morphological features provide basic information for species identification. Studies on the micromorphology of the genus *Pterocarya* are lacking, especially concerning the type of trichomes [37]. The present study provides additional identifying characteristics and, for the first time, highlights the importance of trichomes, as well as the morphology of bracts on male and female flowers, for the differentiation of *Pterocarya* species. When the RAD-seq phylogenetic tree data are combined with the morphological characteristics summarized in FOC [34] and Kozlowski et al. (2018) [37], an in-depth speciation analysis and taxonomic treatment for this genus can be performed.

The species of *Pterocarya* have a number of unifying characteristics, such as large two-winged nutlets and a chambered pith [37]. Moreover, our study revealed that all the *Pterocarya* taxa have peltate trichomes (Figure 5). These common features reflect the close affinities among the species and confirm a monophyletic origin of the genus. The presence or absence of terminal buds and lacunae in the nutlet walls provide two main characteristics for differentiating the two sections and thus support the RAD-seq phylogenetic tree. The phylogenetic framework of *Pterocarya* obtained in this study provides an opportunity to analyze of the evolutionary history of related traits used for the delimitations of different sections and species (Figure 5).

On the basis of our results, we hypothesize that the odd-pinnate leaves represent the ancestral character state, whereas even-pinnate leaves represent the derived character states (Figure 4). Additionally, the close relationship between *P. stenoptera* and *P. tonkinensis* is confirmed by only one morphological feature (winged rachises in *P. stenoptera* but wingless rachises in *P. tonkinensis*) that virtually differentiates them (Figure 4).

Two micromorphological features are very important for distinguishing *P. macroptera* from other species, as well as for differentiating among its varieties (Figure 4). First, both female and male flower bracts in all the varieties of this taxon are tomentose, which is exclusive to *P. macroptera* (all the other species have glabrous bracts). The second important feature is the type of trichomes on mature leaves, which, in *P. macroptera* var. *delavayi*, differs from the type of the other two varieties and confirms the phylogenetic resolution within *P. macroptera*. We have concluded that *P. macroptera* var. *delavayi* should be treated morphologically and phylogenetically as a separate species (*Pterocarya delavayi*). In contrast, the lack of resolution of the phylogenetic tree (Figure 3) and the lack of differences in all the morphological features (Figure 4) suggest that the remaining two varieties (*insignis* and *macroptera*) should be merged into one taxon (*P. macroptera*).

On the basis of these morphological and phylogenetic results, we propose that *Pterocarya* should be divided into six species: three (*P. rhoifolia*, *P. macroptera*, and *P. delavayi*) in sect. *Platyptera* and three (*P. fraxinifolia*, *P. hupehensis*, and *P. stenoptera*) in sect. *Pterocarya*. A new identification key for both sections and all species is provided in the Supplementary Material (Doc. S1).

### 3.3. East Asian versus Southern European/West Asian Disjunctions of Relict Trees: The Importance of the Gobi Desert’s Formation and Climatic Cooling after the Middle Miocene Epoch

East Asia and southern Europe/West Asia (or southern Caucasus and the Mediterranean regions) served as the most important refugia of relict trees during previous climatic fluctuations [23,56,57]. There are many Cenozoic relict woody genera that exhibit the pronounced disjunct distribution patterns between East Asia and southern Europe/West Asia, e.g., *Parrotia*, *Liquidambar*, *Acer*, *Albizia*, *Buxus*, *Carpinus*, *Fagus*, *Diospyros*, *Hippophae*, *Sorbus*, *Taxus*, and *Zelkova* [20,37,58,59]. However, the times and processes leading to the East Asian versus southern European/West Asian disjunct pattern are poorly understood. The results of our study suggest that such a disjunction in the genus *Pterocarya* (sect. *Pterocarya*) occurred during the middle of the Miocene period (15.7 Ma), whereas other studies have suggested different divergence times in other relict genera (e.g., 7.5 Ma for the two species of *Parrotia* in the late Miocene) [20].

The estimated timescale described in our study for the genus *Pterocarya* is in agreement with the known fossil evidence. Fossil records indicate the wide distribution of this genus in Eurasia during the early Neogene period. The absence of fossil data in western Siberia after the Miocene period indicates the disappearance of *Pterocarya* during this period. We hypothesize that the local disappearance of *Pterocarya* in the high latitudes of western Siberia may have been the result of a sharp decrease in global temperatures during the middle Miocene period followed by a major ice sheet expansion from the Arctic [53]. This climatic change may have caused the extinction of *Pterocarya*, along with other relict woody genera, in large parts of western Eurasia and the formation of the isolated refugium in the southern Caucasus and Hyrcanian forests [37].

**Figure 6:**
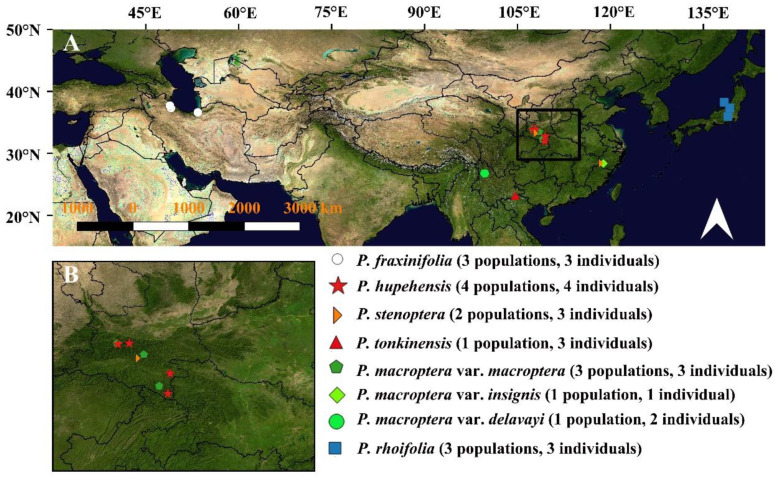
Sampling sites used in this study covering the entire distribution area of *Pterocarya* from the southern Caucasus and East Asia.

The second important event was the desertification of the central Asiatic region and, in particular, the formation of the Gobi Desert. The timing and processes leading to the formation of this desert are still debated [60]. However, recent studies indicate that desertification had already started in the early Miocene period [61,62,63,64]. The results of our study support this hypothesis by indicating that biological exchanges between eastern and western Eurasia may have been restricted during the early and middle Miocene periods (Figure 6).

In our opinion, these two important climatic and geological events (e.g., cooling of the Siberian region and desertification of Central Asia) could have been responsible for the formation of the current western versus eastern Eurasia disjunct distribution pattern within the *Pterocarya* genus, as well as in many other relict tree genera, since the middle Miocene period.

### 3.4. Late Miocene Diversification in the East Asian Refugium

With more than 600 endemic genera of the so-called Arcto-Tertiary flora, East Asia is the main refugium for plants, including numerous emblematic relict tree genera and species, such as *Gingko*, *Davidia*, and *Tetracentron* [29]. Toward the late Miocene period, numerous relict tree genera experienced intense diversification and divergence in the region. A prominent example is the split between two species within *Cercidiphyllum* at the Miocene/Pliocene boundary [29] and the divergence between Chinese *Euptelea pleiosperma* and Japanese *E. polyandra* in the late Miocene period (5.5 Ma) [65]. Recently, the same pattern was detected in Asian butternut (*Juglans* section *Cardiocaryon*), the sister genus of *Pterocarya* in Juglandaceae [30]. In addition, our study confirms this biogeographical pattern. In sect. *Platyptera*, the divergence between *P. rhoifolia* (endemic to Japan) and *P. macroptera* (endemic to China) was estimated to have occurred during the late Miocene period (10.17 Ma). These estimated divergence times are very similar to the divergence times of Asian butternuts (10.9 Ma) [30]. The overlapping distributions of *P. rhoifolia* and *Juglans ailantifolia* in Japan and of *P. macroptera* and *J. cathayensis* in China confirm this hypothesis.

Interestingly, in the East Asia, members of the sect. *Pterocarya* in this genus *P. hupehensis* diverged from *P. stenoptera*/*P. tonkinensis* clade at exactly the same time in the late Miocene period (10.0 Ma). *Pterocarya hupehensis* is restricted to the mountainous areas of southern East Asia, whereas members of the *P. stenoptera*/*P. tonkinensis* clade are widely distributed in eastern and southeastern Asia. The evolutionary history of this clade is clearly in need of further population genetic studies. In the future, new emerging molecular methods (e.g., comparative phylogenomics) based on increased numbers of taxa and sampled populations will help to elucidate the detailed evolutionary and population demographic histories of the genus *Pterocarya*, as well as other relict genera of the SJFR.

## 4. Materials and Methods

### 4.1. Taxon Sampling and DNA Extraction

In this study, we used twenty-two individuals that represented all species and varieties and covered the entire range of each of the species (*n*: number of individuals, *p*: population number): *P. fraxinifolia* (*n* = 3, *p* = 3), *P. hupehensis* (*n* = 4, *p* = 4), *P. macroptera* var. *delavayi* (*n* = 2, *p* = 1), *P. macroptera* var. *insignis* (*n* = 1, *p* = 1), *P. macroptera* var. *macroptera* (*n* = 3, *p* = 3), *P. rhoifolia* (*n* = 3, *p* = 3), *P. stenoptera* (*n* = 3, *p* = 2), and *P. tonkinensis* (*n* = 3, *p* = 1) (Figure 6). *Juglans mandshurica* and *Cyclocarya paliurus* were used as outgroups. The voucher specimens are housed in the herbarium of the Shanghai Chenshan Botanical Garden (CSH), at Niigata University, and at Tarbiat Modares University (TMU). None of the field collections of *Pterocarya* species required specific permissions or involved endangered or threatened species.

DNA extraction was performed with a Qiagen DNeasy Plant Tissue Kit from silica-gel dried leaves according to the manufacturer’s (Qiagen, Valencia, CA, USA) standard protocol. The DNA extraction quality was checked by 1% agarose gel in conjunction with 1 KB Plus DNA Ladder (Invitrogen) or a New England Biolabs 100 bp DNA ladder marker (Ipswich, MA, USA). The genomic DNA concentrations were subsequently quantified with a dsDNA HS kit on a Qubit 2.0 Fluorometer.

### 4.2. RAD-seq Library Preparation

Library preparation and sequencing of the RAD markers from genomic DNA were performed by Majorbio (Shanghai, China) using the restriction enzyme *TaqαI*. The Illumina HiSeqTM platform and an Illumina PE150 were used for sequencing, generating 300~500 bp paired-end reads (P1 and P2). The restriction sites and barcodes were trimmed from each sequence, and bases with FASTQ quality scores below a given value (<20) were replaced with N. Sequences with more than 10% of Ns were discarded. Illumina adapters and sequences smaller than 25 bp were removed, and roughly filtered reads of each individual were obtained.

### 4.3. Processing and Clustering RAD-seq Data

After receiving the sequencing data, we demultiplexed and processed the roughly filtered reads using the software pipeline IPYRAD v0.7.11 [66]. Nucleotide bases with a Phred quality score (Q) below 33 were replaced with an ambiguous base (“N”), and reads with more than 5% “N”s were discarded. Filtered reads of each individual were first assembled de novo into putative loci. For within-sample clustering, the sequences were clustered at 0.85 similarity by VSEARCH [67]. After clustering, the rates of heterozygosity (H) and sequencing errors (E) were jointly estimated from aligned clusters for each sampled individual [68], and the average parameter values were used when calling consensus bases. Loci containing more than two alleles after error correction were excluded as potential paralogs since *Pterocarya* species are diploid [42]. Consensus sequences were then aligned with Muscle v3.8.31 [69]. A final filtering step excluded any loci containing one or more sites that appeared heterozygous across more than five samples, as such loci may represent a fixed difference among clustered paralogs rather than a true heterozygous site at the broad phylogenetic scale. The remaining clusters representing multiple alignments of putative orthologs were treated as RAD-seq loci and assembled into phylogenetic data matrices.

### 4.4. Morphological Evaluations and Data Sets

During our fieldwork between 2014 and 2016, we collected all eight taxa of *Pterocarya* and the two outgroups. Afterwards, we evaluated all the morphological characteristics (including the trunks, bark, buds, leaves, flowers, and fruits) as described in *FOC* and other publications [33,34,37]. Due to the absence of leaf epidermal features in the literature, we studied the trichomes of all species and varieties of the mature leaves. The dried materials were directly mounted onto stubs without any treatment. After being sputter coated with gold, the specimens were examined and imaged via scanning electron microscopy (SEM) (Quanta 250). The descriptions and terminology of the trichomes mainly followed those of Deng et al. (2014) [70].

We collected data on 13 total binary morphological characteristics: terminal buds (naked or scaled), lacunae in the walls on nutlets (presence or absence), bud-scale scars on branchlets (presence or absence), position of male spike (old or new growth), pinnate leaves (odd or even), fruit wings (linear or semi- or bi-cular), angle of fruit wings (<90° or approximately 180°), solitary trichomes (presence or absence), number of leaflets (5–13 or 11–27), fasciculate trichomes (presence or absence), morphology of bracts of flowers (glabrous or tomentose), morphology of leaf abaxial surface (glabrous or tomentose), and rachises (wingless or winged). These characteristics were easy to identify and can be treated as binary and were stated on our molecular phylogenetic tree.

### 4.5. Phylogenetic Reconstruction

The single end of the paired-end reads (P1) of RAD-seq data was used for phylogenetic inference and all the data were submitted to GenBank with information related to taxonomy and GenBank accession numbers (Table S1). Maximum likelihood (ML) and Bayesian Inference (BI) trees were inferred using RAxML v8.2.4 [71] and MrBayes v3.2.6 [72], respectively. An ML tree with random starting trees and a GTR + GAMMA nucleotide substitution model was constructed, and the reliability of the tree topology was determined by 200 nonparametric bootstrapped replicates. The BI analyses were started with random trees, and four parallel Markov Chain Monte Carlo (MCMC) searches were performed for 100 million generations each. The trees were sampled every 100 generations, and the first 20% of each run was discarded as burn-in. Tracer v1.6 [73] was used to check the log-likelihood of sampled trees and determine when stationarity had been reached.

### 4.6. Fossil Constraints and Estimations of Divergence Times

Two fossils were used as minimum age constraints for two nodes. The first fossil was *Juglans clarnensis*, which was identified as the oldest *Juglans* fossil and dated back to the Eocene epoch (34–55 Ma) in North America [36,37]. The second fossil was *Pterocarya smileyi* from North America. This fossil dated back to the Miocene epoch (5.3–23.0 Ma) and was the first fossil identified as having an affinity with section *Pterocarya* [36]. To infer divergence times, a relaxed clock model was analyzed under a MCMC simulation in BEAST v1.7.5 [74]. A prior Yule tree was used with an uncorrelated lognormal molecular clock. Tree and log files were generated from two runs with different starting seeds. The MCMC length was 100 million generations, with parameter sampling occurring every 1000 generations. Convergence was assessed by Tracer v1.6 [73], and the effective sample sizes (ESSs) of all the parameters were also assessed. A maximum clade credibility (MCC) tree was generated by TreeAnnotator v1.7.4 after the first 20% of the trees had been removed as burn-in [74].

## Figures and Tables

**Table 1:** Summary of RAD-seq data processing (paired-end reads) from 24 samples used in this study.

Summary Statistic	Raw Reads	Clean Reads	Total Length of Clean Reads (Gbp)	Clean Data Percentage (%)	Q30 Percentage (%)	GC Percentage (%)
Average	11,055,000	9,947,083	1.54	84.30	91.54	47.10
Maximum	18,580,000	17,290,000	2.36	90.02	92.39	58.57
Minimum	4,780,000	3,790,000	0.69	76.16	88.55	43.03
SD	3,232,889	3,250,324	0.37	3.64	0.88	3.91

**Table 2:** Summary statistics of filtering and clustering results of one single end RAD sequences (R1) from 24 samples used for the phylogenetic analysis in this study.

Summary Statistic	RAD Tags (R1)	Total Clusters (R1)	Mean Depth of Clusters	H	E	Consensus Loci	Loci in Final Data Set
Average	5,502,955	1,728,343	15.67	0.0413	0.0103	102,981	9287
Maximum	8,591,043	3,985,579	17.75	0.0526	0.0136	204,925	13,650
Minimum	2,495,755	769,873	13.04	0.0350	0.0075	38,695	4222
SD	1,316,019	733,868	1.11	0.0043	0.0016	39,940	2668

## Supplementary Materials

The following are available online at https://www.mdpi.com/2223-7747/9/11/1524/s1, Figure S1: Bayesian Inference (BI) tree of *Pterocarya*. PMM: *P. macroptera* var. *macroptera*, PMI: *P. macroptera* var. *insignis*, and PMD: *P. macroptera* var. *delavayi*, Table S1: List of taxa included in dataset of restriction site-associated DNA sequencing (RAD-seq) for the phylogenetic analysis of *Pterocarya* with information related to taxonomy and GenBank accession numbers, Table S2: Detail information of RAD-seq data processing for *Pterocarya* used in this study, Doc. S1: Identification key for the sections and species in the genus *Pterocarya* (Juglandeceae).
