# Supplementary material for: Phylogeny, Taxonomy, and Biogeography of Pterocarya (Juglandaceae)
Source: Plants (Basel). 2020 Nov 9;9(11):1524. doi: 10.3390/plants9111524 (PMC7696814; doi:10.3390/plants9111524)
Supplement: Supplementary file 1 [file plants-09-01524-s001.zip › plants-972597-supplementary/Supplementary Files/Doc. S1. Identification key for the sections and species in the genus Pterocarya.docx]

**Doc. S1. Identification key for the sections and species in the genus *Pterocarya* (Juglandeceae)**

1a. Terminal bud with 2-5 caducous scales; male spike lateral at base of new growth; nutlets without lacunae in wall. (Section ***Platyptera***).........................................................................................2

1b. Terminal bud naked; male spike lateral on old growth or scattered on new growth; nutlets with large lacunae in wall. (Section ***Pterocarya***) ..............................................................................3

2a. Leaflets 11-21; bract on male and female flower with gland; axis of fruiting spike pubescent; nutlets glabrous ................................................................................................ ***P. rhoifolia***

2b. Leaflets 7-13; bract on male and female flower with tomentose; abaxial leaf surface (matured leaves) glabrous or only fasciculate trichomes along main vines .................... ***P. macroptera***

2c. Leaflets 7-13; bract on male and female flower with tomentose; abaxial leaf surface (matured leaves) densely covered with solitary trichomes .............................................. ***P. delavayi***

3a. Leaves odd-pinnate ......................................................................................................................4

3b. Leaves even-pinnate ....................................................................................................................5

4a. Leaflets 11-21; nutlets wings broad, elliptic-ovate ....................................... ***P. fraxinifolia***

4b. Leaflets 5-11; nutlets wings broad, elliptic-ovate ........................................ ***P. hupehensis***

5a. Leaflets 6-22; nutlets wings narrow to linear; rachis usually winged ......... ***P. stenoptera***

5b. Leaflets 6-22; nutlets wings narrow to linear; rachis wingless ***P. stenoptera* var. *tonkinensis***

***Pterocarya rhoifolia*** Siebold & Zuccarini, Fl. Jap. 141. 1845. = *P. sorbifolia* Sieb. et Zucc., l. c.

***Pterocarya macroptera*** Batalin, Trudy Imp. S.-Peterburgsk. Bot. Sada 13: 100. 1893. = *P. insignis* Rehder & E. H. Wilson in Sargent, Pl. Wilson. 3: 183. 1916. = *P. macroptera* var. *insignis* (Rehder & E. H. Wilson) W. E. Manning, Bull. Torrey Bot. Club 102: 165. 1975.

***Pterocarya delavayi*** Franch. in Journ. de Bot. 12: 317. 1898. = *P. macroptera* var. *delavayi* (Franchet) W. E. Manning, Bull. Torrey Bot. Club 102: 165. 1975. = *P. forrestii* W. W. Smith. in Not. Bot. Gard. Edinb. 14: 87, 261, 317.1924.

***Pterocarya fraxinifolia*** (Poir.) Spach, 1834.

***Pterocarya hupehensis*** Skan in F. B. Forbes & Hemsley, J. Linn. Soc., Bot. 26: 493. 1899. = *Pterocarya sprengeri* Pampan. in Nouv. Giorn. Bot. Ital. n. s. 22: 274. 1915.

***Pterocarya stenoptera*** C. de Candolle, Ann. Sci. Nat., Bot., Ser. 4, 18: 34. 1862. = *Acer mairei* H. Leveille., l. c. pro. syn. = *P. chinensis* Lavallee, l. c. pro. syn. = *P. esquirollii* Levl., Cat. Pl. Yunn. 135. 1916. = *P. japonica* Dipp., Handb. Laubhk. 2: 329. 1892. = *P. japonica* Lavallee, l. c. pro. syn. = *P. laevigata* Lavallee, En. Arb. Segr. 217. 1877, pro. syn. = *P. stenoptera* C. DC. var. *brevialata* Pampan. in Nouv. Giorn. Bot. Ital. n. s. 22: 274. 1915. = *P. stenoptera* C. DC. var. *kouitchensis* Franch. l. c. 318. = *P. stenoptera* C. DC. var. *sinensis* Graebner. in Mitt. Deutsch. Ges. no. 20: 215. 1911. = *P. stenoptera* C. DC. var. *typica* Franch. in Journ. de Bot. 12: 317. 1898.

***Pterocarya stenoptera* var. *tonkinensis*** (Franchet) Dode, Bull. Soc. Dendrol. France 70: 67. 1929. = *P. stenoptera* C. DC. var. *tonkinensis* Franch. in Journ. Bot. Morot. 318. 1898.
