# Supplementary material for: Phylogeny, Taxonomy, and Biogeography of Pterocarya (Juglandaceae)
Source: Plants (Basel). 2020 Nov 9;9(11):1524. doi: 10.3390/plants9111524 (PMC7696814; doi:10.3390/plants9111524)
Supplement: Supplementary file 1 [file plants-09-01524-s001.zip › plants-972597-supplementary/Supplementary Files/Figure S1 Bayesian Inference tree of Pterocarya.docx]

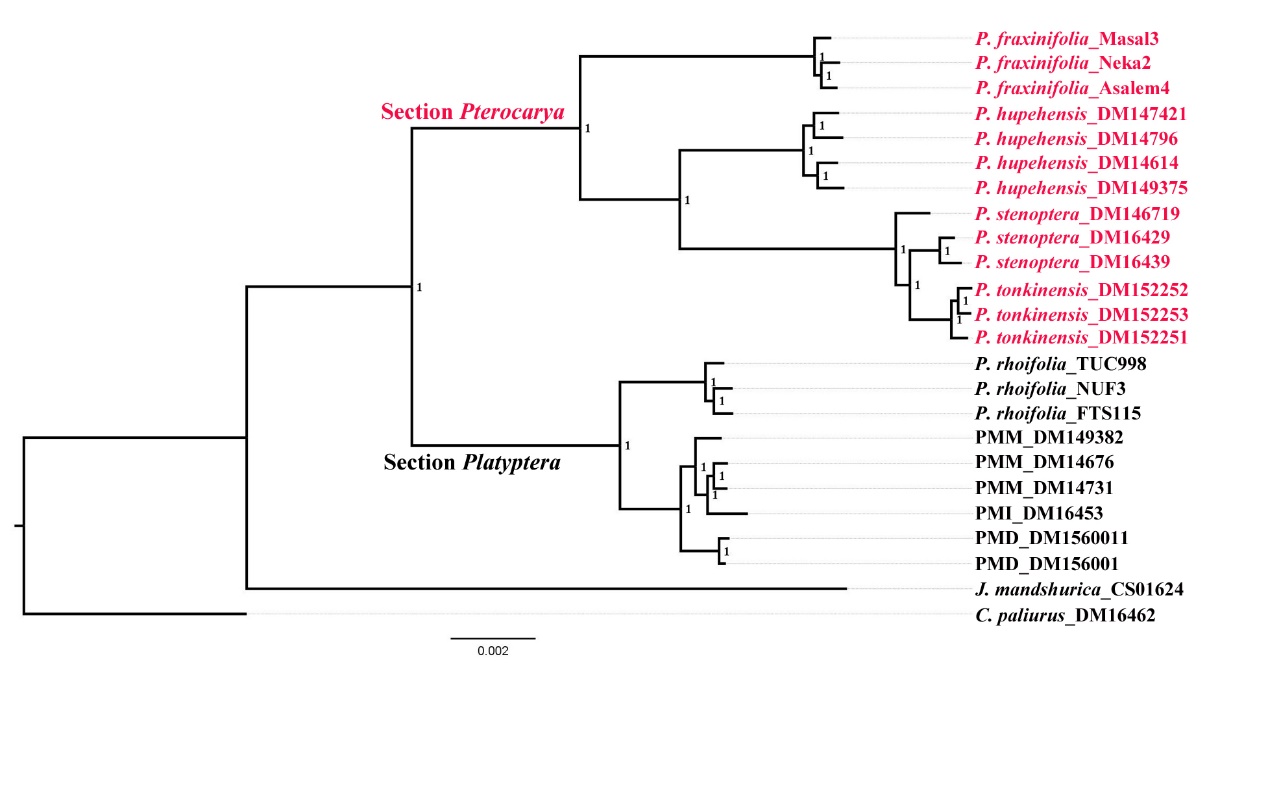


**Figure S1** Bayesian Inference (BI) tree of *Pterocarya*. PMM: *P. macroptera* var. *macroptera*, PMI: *P. macroptera* var. *insignis*, and PMD: *P. macroptera* var. *delavayi*.
