# Supplementary material for: Phylogeny, Taxonomy, and Biogeography of Pterocarya (Juglandaceae)
Source: Plants (Basel). 2020 Nov 9;9(11):1524. doi: 10.3390/plants9111524 (PMC7696814; doi:10.3390/plants9111524)
Supplement: Supplementary file 1 [file plants-09-01524-s001.zip › plants-972597-supplementary/Supplementary Files/Table S1 List of taxa with GenBank accession numbers for RAD-seq data.docx]

Table S1. List of taxa included in dataset of restriction site-associated DNA sequencing (RAD-seq) for the

phylogenetic analysis of *Pterocarya* with information related to taxonomy and GenBank accession numbers.

| **Species** | **specimen vouchers** | **GenBank accession number** |
| --- | --- | --- |
| *Pterocarya fraxinifolia* | Asalem4 | SAMN15793765 |
| *Pterocarya fraxinifolia* | Neka2 | SAMN15793766 |
| *Pterocarya fraxinifolia* | Masal3 | SAMN15793767 |
| *Pterocarya hupehensis* | DM14614 | SAMN15793768 |
| *Pterocarya hupehensis* | DM14937_5 | SAMN15793769 |
| *Pterocarya hupehensis* | DM14742_1 | SAMN15793770 |
| *Pterocarya hupehensis* | DM14796 | SAMN15793771 |
| *Pterocarya stenoptera* | DM14671_9 | SAMN15793772 |
| *Pterocarya stenoptera* | DM16429 | SAMN15793773 |
| *Pterocarya stenoptera* | DM16439 | SAMN15793774 |
| *Pterocarya tonkinensis* | DM15225_1 | SAMN15793775 |
| *Pterocarya tonkinensis* | DM15225_2 | SAMN15793776 |
| *Pterocarya tonkinensis* | DM15225_3 | SAMN15793777 |
| *Pterocarya macroptera* var. *macroptera* | DM14676 | SAMN15793778 |
| *Pterocarya macroptera* var. *macroptera* | DM14731 | SAMN15793779 |
| *Pterocarya macroptera* var. *macroptera* | DM14938_2 | SAMN15793780 |
| *Pterocarya macroptera* var. *insignis* | DM16453 | SAMN15793781 |
| *Pterocarya macroptera* var. *delavayi* | DM15600_1 | SAMN15793782 |
| *Pterocarya macroptera* var. *delavayi* | DM15600_11 | SAMN15793783 |
| *Pterocarya rhoifolia* | FTS115 | SAMN15793784 |
| *Pterocarya rhoifolia* | NUF3 | SAMN15793785 |
| *Pterocarya rhoifolia* | TUC998 | SAMN15793786 |
| *Juglans mandshurica* | CS01624 | SAMN15793787 |
| *Cyclocarya paliurus* | DM16462 | SAMN15793788 |
