# Supplementary material for: Phylogeny, Taxonomy, and Biogeography of Pterocarya (Juglandaceae)
Source: Plants (Basel). 2020 Nov 9;9(11):1524. doi: 10.3390/plants9111524 (PMC7696814; doi:10.3390/plants9111524)
Supplement: Supplementary file 1 [file plants-09-01524-s001.zip › plants-972597-supplementary/Supplementary Files/Table S2 Detail information of RAD-seq data processing for Pterocarya used in this study.docx]

**Table S2** Detail information of RAD-seq data processing for *Pterocarya* used in this study.

| Taxa | Voucher number | Raw reads | Clean reads | Total length of clean reads (Gbp) | Clean data percentage (%) | Q30 percentage (%) | GC percentage (%) | RAD tags (R1) | Total clusters (R1) | Mean depth of clusters | H | E | Consensus loci | Loci in final data set |
| --- | --- | --- | --- | --- | --- | --- | --- | --- | --- | --- | --- | --- | --- | --- |
| *P. fraxinifolia* | Asalem4 | 7800000 | 6510000 | 1.1 | 79.07 | 91.77 | 44.35 | 3942095 | 1080111 | 16.58 | 0.04099 | 0.010828 | 73341 | 8476 |
| *P. fraxinifolia* | Neka2 | 11570000 | 10650000 | 1.49 | 87.09 | 92.04 | 44.69 | 5290757 | 1134365 | 16.78 | 0.039965 | 0.009115 | 117953 | 10682 |
| *P. fraxinifolia* | Masal3 | 4780000 | 3790000 | 0.69 | 76.16 | 91.89 | 45.21 | 2495755 | 769873 | 17.54 | 0.040667 | 0.011686 | 38695 | 5615 |
| *P. hupehensis* | DM14614 | 11490000 | 9810000 | 1.37 | 80.19 | 92.33 | 43.86 | 4856374 | 1336245 | 14.55 | 0.04112 | 0.010205 | 105389 | 11694 |
| *P. hupehensis* | DM14937-5 | 13300000 | 12100000 | 1.66 | 84.43 | 90.9 | 50.09 | 5995676 | 2186747 | 15.53 | 0.043706 | 0.011905 | 83582 | 8601 |
| *P. hupehensis* | DM14742-1 | 11540000 | 10590000 | 1.48 | 86.64 | 92.27 | 43.32 | 5254300 | 1292504 | 15.45 | 0.039872 | 0.009336 | 121900 | 12495 |
| *P. hupehensis* | DM14796 | 9270000 | 8070000 | 1.42 | 82.97 | 91.72 | 46.88 | 5097271 | 1547982 | 16.64 | 0.041393 | 0.01008 | 90084 | 9828 |
| *P. stenoptera* | DM14671-9 | 8740000 | 7830000 | 1.24 | 84.44 | 92.08 | 44.95 | 4440500 | 1307072 | 14.2 | 0.036869 | 0.009983 | 100946 | 11228 |
| *P. stenoptera* | DM16429 | 14860000 | 13680000 | 1.94 | 87.96 | 92.39 | 43.03 | 6804761 | 1388435 | 15.87 | 0.034994 | 0.007524 | 195671 | 13650 |
| *P. stenoptera* | DM16439 | 15740000 | 14630000 | 2.08 | 89.14 | 92.34 | 43.81 | 7283377 | 1549986 | 15.66 | 0.0367 | 0.007555 | 204925 | 12948 |
| *P. tonkinensis* | DM15225-1 | 8180000 | 6810000 | 1.22 | 79.08 | 91.3 | 47.35 | 4388477 | 1350621 | 14.56 | 0.037667 | 0.009526 | 91516 | 10111 |
| *P. tonkinensis* | DM15225-2 | 8850000 | 7660000 | 1.35 | 83.01 | 92.19 | 44.6 | 4808264 | 1299253 | 14.85 | 0.039095 | 0.009374 | 110833 | 11782 |
| *P. tonkinensis* | DM15225-3 | 8520000 | 7540000 | 1.3 | 84 | 91.39 | 49.48 | 4659310 | 1675749 | 15.61 | 0.039962 | 0.008784 | 75382 | 8681 |
| *P. macroptera* var. *macroptera* | DM14676 | 7240000 | 6230000 | 1.16 | 81.21 | 90.46 | 52.28 | 4196524 | 1425021 | 17.75 | 0.041569 | 0.011081 | 61113 | 5869 |
| *P. macroptera* var. *macroptera* | DM14731 | 14590000 | 13480000 | 1.84 | 85.1 | 90.33 | 52.43 | 6679899 | 3039846 | 15.69 | 0.050038 | 0.013615 | 57590 | 5467 |
| *P. macroptera* var. *insignis* | DM16453 | 12840000 | 12010000 | 1.69 | 88.72 | 91.92 | 45.66 | 5968752 | 1803293 | 14.72 | 0.041227 | 0.009496 | 129954 | 10868 |
| *P. macroptera* var. *macroptera* | DM14938-2 | 10090000 | 9070000 | 1.5 | 84.85 | 91.28 | 45.91 | 5404859 | 1676945 | 15.86 | 0.044306 | 0.011414 | 91893 | 9029 |
| *P. macroptera* var. *delavayi* | DM15600-1 | 10740000 | 9930000 | 1.74 | 86.58 | 90.89 | 52.64 | 6260018 | 2713422 | 15.14 | 0.047639 | 0.013255 | 63517 | 6680 |
| *P. macroptera* var. *delavayi* | DM15600-11 | 18580000 | 17290000 | 2.36 | 86.27 | 88.55 | 58.57 | 8591043 | 3985579 | 13.04 | 0.052552 | 0.011085 | 81397 | 4222 |
| *P. rhoifolia* | FTS115 | 14030000 | 13320000 | 1.87 | 90.02 | 92.06 | 46.37 | 6623769 | 1945248 | 15.78 | 0.038889 | 0.009253 | 138867 | 11317 |
| *P. rhoifolia* | NUF3 | 14450000 | 13490000 | 1.9 | 89.04 | 92.14 | 44.74 | 6715044 | 1650985 | 17.23 | 0.037913 | 0.008396 | 150035 | 12114 |
| *P. rhoifolia* | TUC998 | 10880000 | 9560000 | 1.89 | 83.63 | 90.83 | 51.18 | 6808079 | 2768757 | 15.28 | 0.04592 | 0.011516 | 87735 | 7792 |
| *Juglans mandshurica* | CS01624 | 8600000 | 7500000 | 1.35 | 83.43 | 91.96 | 44.31 | 4826057 | 1166206 | 16.57 | 0.037692 | 0.009449 | 108570 | 6841 |
| *Cyclocarya paliurus* | DM16462 | 8640000 | 7180000 | 1.31 | 80.12 | 91.89 | 44.77 | 4679964 | 1385980 | 15.08 | 0.04015 | 0.011711 | 90660 | 6893 |
